# Implication of Paris Agreement in the context of long-term climate mitigation goals

**DOI:** 10.1186/s40064-016-3235-9

**Published:** 2016-09-20

**Authors:** Shinichiro Fujimori, Xuanming Su, Jing-Yu Liu, Tomoko Hasegawa, Kiyoshi Takahashi, Toshihiko Masui, Maho Takimi

**Affiliations:** 1 Center for Social and Environmental Systems Research, National Institute for Environmental Studies (NIES), National Institute for Environmental Studies, 16-2 Onogawa, Tsukuba, Ibaraki 305–8506 Japan; 2 International Institute for Applied Systems Analysis, Schlossplatz-1, Laxenburg, 2361 Austria; 3 Mizuho Information and Research Institute, Inc., 2-3 Kanda-Nishikicho, Chiyoda-ku, Tokyo 101-8443 Japan

**Keywords:** AIM, Integrated assessment model, Land use, Climate change mitigation

## Abstract

The Paris Agreement confirmed the global aim to achieve a long-term climate goal, in which the global increase in mean temperature is kept below 2 °C compared to the preindustrial level. We investigated the implications of the near-term emissions targets (for around the year 2030) in the context of the long-term climate mitigation goal using the Asia-Pacific Integrated Model framework. To achieve the 2 °C goal, a large greenhouse gas emissions reduction is required, either in the early or latter half of this century. In the mid-term (from 2030 to 2050), it may be necessary to consider rapid changes to the existing energy or socioeconomic systems, while long-term measures (after 2050) will rely on the substantial use of biomass combined with carbon capture and storage technology or afforestation, which will eventually realize so-called negative CO_2_ emissions. With respect to the policy context, two suggestions are provided here. The first is the review and revision of the nationally determined contributions (NDCs) in 2020, with an additional reduction target to the current NDCs being one workable alternative. The second suggestion is a concrete and numerical mid-term emissions reduction target, for example to be met by 2040 or 2050, which could also help to achieve the long-term climate goal.

**Electronic supplementary material:**

The online version of this article (doi:10.1186/s40064-016-3235-9) contains supplementary material, which is available to authorized users.

## Background

The Conference of the Parties (COP) 21 to the United Nations Framework Convention on Climate Change (UNFCCC) adopted the Paris Agreement (PA) (United Nations Framework Convention on Climate Change 2015). The PA provides a framework for global action to address climate change in the period after 2020. The objective of the agreement is to maintain the increase in global temperatures well below 2 °C above preindustrial levels.

The PA requires Parties to prepare nationally determined contributions (NDCs), indicating an individual country’s emissions reduction commitments, take measures to achieve their objectives, and report on progress. To raise the level of ambition over time, Parties must submit updated NDCs every 5 years. Each Party’s new NDC must be more ambitious than its previous NDC. Over 180 Parties to the UNFCCC communicated their intended nationally determined contributions (INDCs) for 2025/2030 before COP21.

There have already been several assessments related to INDCs published either in scientific papers, websites, or reports (Meinshausen et al. 2015; Fawcett et al. 2015; Gokul et al. 2015; International Energy Agency 2015; Climate Action Tracker 2015; Lomborg 2015; Benveniste et al. 2015; Kitous and Keramidas 2015; Hof et al. 2015). Some have proposed alternative scenarios to achieve the 2 °C goal, because INDCs based on emissions have led to scenarios with a temperature rise larger than 2 °C. Some assessments have made comparisons with the recent Fifth Assessment Report of Intergovernmental Panel on Climate Change (IPCC AR5) scenario database (International Institute for Applied Systems Analysis 2015a), and made allocations based on the multiple effort sharing schemes. The consensus among the assessments at this stage is that current INDCs are not in line with the 2 °C goal, which was also stated in the PA (United Nations Framework Convention on Climate Change 2015). To achieve the 2 °C goal, either a further emissions reduction before 2030, or more drastic and quick reductions after this date are required.

The difficulties in reducing greenhouse gas (GHG) emissions after 2030 are not obvious. Therefore, we investigated two issues with respect to the relationship between INDCs and the long-term climate mitigation goal. The first is what difficulties exist in realizing the 2 °C goal under the current INDCs. We considered difficulties that might arise in the medium and long term. Here, we defined medium and long term as from 2030 to 2050, and after 2050, respectively. The second issue is what policy suggestions can be derived from such assessments. Regarding the first issue, we also considered the modeling limitations in the current integrated assessment model.

To address these issues, we conducted a scenario assessment using the Asia-Pacific Integrated Model (AIM) framework. The remainder of this paper is organized by follows. section “Methods” provides the overall methodology, as well as the model and scenario framework. In section “Results”, the results are presented and analyzed. An interpretation of the results and limitations of the study are provided in section “Discussion”, and the conclusions are presented in section “Conclusions”.

## Methods

**Fig. 1:**
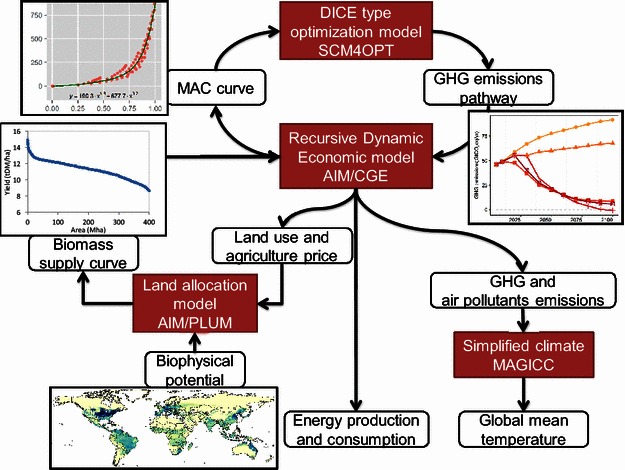
Asia-Pacific Integrated Model (AIM) modeling framework

Figure 1 shows the overall modeling framework. Several models exchanged information with each other within this framework, and the core of the simulation was a global computable general equilibrium model: AIM/Computable General Equilibrium (CGE). AIM/CGE produces a marginal abatement cost (MAC) curve by experimenting with specific carbon price pathway scenarios (e.g., 3 % per year increase). This information was used for the calibration of the MAC function (the actual parameter adjusted by this information is the reduction control rate) in a Dynamic Integrated model of Climate and the Economy (DICE) type intertemporal optimization model (Nordhaus and Sztorc 2013)—the Simple Climate Model for Optimization (SCM4OPT). SCM4OPT was used to produce GHG emissions pathways under particular climate constraints for AIM/CGE. Then, AIM/PLUM (integration Platform for Land-Use and Environmental Modelling) (Hasegawa et al. under review) was used to conduct a land use allocation of agricultural cropland and pasture land. AIM/PLUM downscales the regionally aggregated land use information provided by AIM/CGE into gridded information. This land use allocation allowed us to obtain biomass energy crop supply curves for each region, which represented the relationship between the bioenergy cropland area and its yield. These supply curves were fed into the AIM/CGE together with emissions constraints from SCM4OPT. Finally, the GHG and air pollutant emissions, which are computed by AIM/CGE, were input to a simplified climate model, the Model for the Assessment of Greenhouse-gas Induced Climate Change (MAGICC) version 6 (Meinshausen et al. 2011), to simulate global mean temperature and radiative forcing under the detailed emissions information produced by AIM/CGE. SCM4OPT could also be used for the final climate estimates, but here we prioritized its comparability with other integrated assessment modeling (IAM) studies, which have predominantly used MAGICC. The simulation covers the period from 2005 to 2100.

### AIM/CGE

The CGE model used in this study is a recursive dynamic general equilibrium model that covers all regions of the world, and is widely used in climate mitigation and impact studies (Hasegawa et al. 2014, 2015a, 2016; Mittal et al. 2016; Fujimori et al. 2014b, c, 2015; Hasegawa et al. 2015b). The main inputs of the model are assumptions about population growth, GDP growth, energy technology efficiency, resource reserves, and related factors. The model classifies the world into 17 geopolitical regions and 42 industrial classifications (shown in the Additional file 1).

It was important that the industrial classification of energy sectors, including power sectors, were disaggregated in detail, because energy systems and technological descriptions were crucial for the purposes of this study. Moreover, to assess bioenergy and land use competition appropriately, agricultural sectors also need to be highly disaggregated (Fujimori et al. 2014a). The details of the model structure and mathematical formulas are given in Fujimori et al. (2012).

The production sectors were assumed to maximize profits under multi-nested constant elasticity substitution (CES) functions and at each input price. Energy transformation sectors input energy and value-added as a fixed coefficient, whereas energy end-use sectors have elasticities between energy and value-added. They were treated in this manner to deal appropriately with energy conversion efficiency in the energy transformation sectors. Power generated from several energy sources was combined by a logit function, although a CES function is often used in other CGE models. We chose this method for the consideration of the energy balance because the CES function does not guarantee a material balance (Schumacher and Sands 2006). Household expenditure on each commodity was described by a linear expenditure system (LES) function. The saving ratio was endogenously determined to balance saving and investment, and capital formation for each item was determined using a fixed coefficient. The Armington assumption was used for trade, and the current account was assumed to be balanced.

In addition to energy-related CO_2_ emissions, CO_2_ from other sources, CH_4_, and N_2_O (including changes resulting from land use and non-energy-related emissions) were included as GHG emissions in this model. Global warming potentials were used when considering emissions of the six gases covered by the Kyoto protocol as specified in the fourth IPCC assessment report (IPCC 2007).

Once an emission constraint was placed on a region, a carbon tax became a complementary variable to that constraint. Such a tax raises the price of fossil fuel goods when emissions are constrained, and promotes energy savings and the substitution of fossil fuels by alternative energy sources with lower emissions. An emissions tax, called the GHG emissions price, is also an incentive to reduce non-energy-related emissions. The revenue from this tax is assumed to go to households.

### AIM/PLUM

The AIM/PLUM is a global land-use allocation model used to downscale the AIM/CGE’s aggregated regional land-use projections into a spatial gridded land-use pattern (0.5° × 0.5°) for the interactive assessment of human activities and biophysical elements. Regional-scale land demand estimated by AIM/CGE (17 regions) was fed into the AIM/PLUM land-use allocation model and was spatially distributed into grid cells (0.5° × 0.5°). The cropland and afforestation area was allocated based on optimization (profit maximization), where a land owner was assumed to decide the mix of land-uses to obtain the highest profit for a given biophysical land productivity condition (e.g., crop yield production per unit area). Because the optimization was solved for each region that had the same regional classification used in AIM/CGE, land transactions across the regions were not allowed. The allocation was conducted in 5-year steps. There were seven crop types, with or without irrigation. Land for harvested wood was excluded from the model framework.

The potential yield of crops was based on estimates from the Lund-Potsdam-Jena managed Land Dynamic Global Vegetation and Water Balance Model (LPJmL) (Bondeau et al. 2007) prepared for Inter-Sectoral Impact Model Inter-comparison Project (ISI-MIP) (Rosenzweig et al. 2014). The bioenergy crop yield and forest carbon sequestration were based on estimates from the Vegetation Integrative Simulator for Trace Gases (VISIT) (Ito and Inatomi 2012). Please see Hasegawa et al. (under review) for more details.

### SCM4OPT

SCM4OPT is an intertemporal optimization model based on DICE2013R, in which the discounted total welfare is maximized under given socioeconomic and climate conditions. The main extension from DICE consisted of two parts. The first part considered anthropogenic emissions. In the original DICE, only industrial CO_2_ emissions are explicitly treated as endogenous emissions, while the rest are exogenously given as radiative forcings. In our extension, we incorporated the major GHGs (land related CO_2_, CH_4_, N_2_O, and F-Gases) and other climate forcers (SOx, NOx, CO, OC, BC, NH_3_, and VOCs). For the baseline case, AIM/CGE emissions were used directly, whereas for the climate policy cases, the MAC curves, which were represented by a function of the carbon price were applied to all gases. These MAC curves were calibrated from the AIM/CGE outcomes.

The second part of the extension was in the climate module. Because many climate forcers are incorporated, the functions from emissions to forcings were added mostly from MAGICC 6.0. Natural forcing and the products of the chemical reactions of emissions were also added (e.g., cloud albedo, stratospheric ozone, tropospheric ozone, stratospheric water–vapor from CH_4_ oxidization, solar and volcanic inputs). For more detail see the Additional file 1.

### Scenarios

We simulated five scenarios in this study, and they are summarized in Table 1. The first scenario was a baseline scenario, which includes no climate policies. A climate policy was defined as the implementation of emissions constraints or a carbon price. The second scenario was the INDCSamePrice scenario, which implemented the Cancun pledge by 2020, and the GHG emissions then followed INDCs targets for individual regions until 2030. How the individual country emissions pledges were placed into the AIM/CGE aggregated regions is detailed in the Additional file 1. The carbon price was assumed to be constant at the 2030 level for all dates afterwards. The remaining three scenarios were intended to stabilize the atmospheric CO_2_ concentration at 450 ppm CO_2_-equivalent after 2100. This concentration stabilization is often considered equivalent to the 2 °C goal, i.e., maintaining the temperature increase from preindustrial level below 2 °C with a probability of >66 %. However, the emissions pathways differed among the three scenarios. The first scenario, 450ppmeRCP, roughly followed the Representative Concentration Pathway 2.6 (RCP2.6) (Vuuren et al. 2011), with a global uniformed carbon price applied after 2015, given the exogenous emissions pathway. The 450ppmeCancunP scenario met the 2020s Cancun pledge, and then reduced emissions using a global uniformed carbon price. The 450ppmeINDC scenario followed the INDC targets, and then reduced emissions with a global uniformed carbon price. The GHG emissions pathways in 450ppmeCancunP and 450ppmeINDC after 2020 and 2030 were determined using SCM4OPT, which intertemporally optimizes the emission levels for each GHG by maximizing the social welfare. While ultimate climate goal of 450ppmeCancunP and 450ppmeINDC scenarios are same, the emissions pathways are different due to the INDC 2030s target, and that eventually differ intermediate mitigation strategy between two scenarios.

**Table 1:** List of scenarios

Scenario name	GHG emissions reduction		
	2015–2020	2020–2030	2030–2100
Baseline	None		
INDCSamePrice	Cancun pledge	INDCs	Same carbon price in 2030
450ppmeRCP	Same as RCP2.6 emissions pathway		
450ppmeCancuunP	Cancun pledge	Equivalent to cumulative emissions in 450ppmeRCP	
450ppmeINDC	Cancun pledge	INDCs	Equivalent to cumulative emissions in 450ppmeRCP

Here, the population and GDP assumptions under Shared Socioeconomic Pathway 2 (SSP2) (International Institute for Applied Systems Analysis 2015b) were used for the drivers of basic human activity and other technological assumptions were based on Fujimori et al. (2016).

## Results

### GHG emissions

**Fig. 2:**
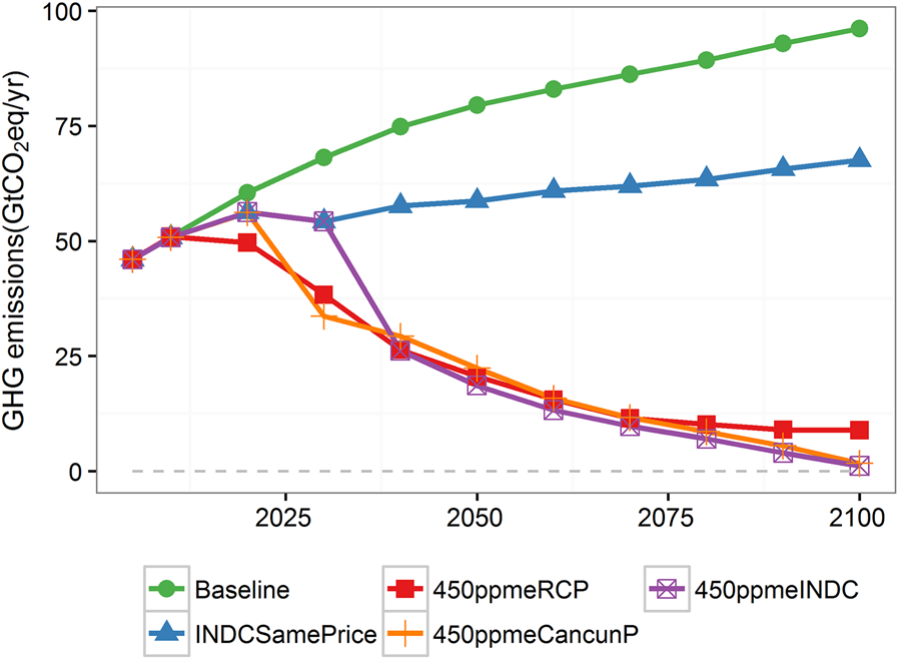
Global total greenhouse gas (GHG) emissions

**Fig. 3:**
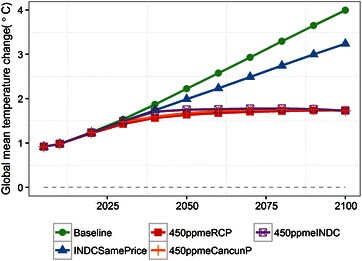
Global mean change in temperature relative to the preindustrial level

GHG emissions (the six gases covered by the Kyoto protocol) are shown in Fig. 2. The GHG emissions at baseline steadily increase during this century due to the population increase and GDP growth. The rate of increase is higher in the first part of the period than the latter part because the GDP growth rate is relatively high in the early period. In 2100, GHG emissions reach around 90 GtCO_2_eq/year. The GHG emissions in INDCSamePrice are slightly lower than at baseline in 2020. In 2030, the emissions are 57 GtCO_2_eq/year, while baseline emissions are 75 GtCO_2_eq/year. After 2030, due to the implementation of the continuous carbon price, the GHG emissions in 2100 are lower than at baseline at 67 GtCO_2_eq/year. The global mean increases in temperature from the preindustrial level in 2100 for baseline and INDCSamePrice are 4.0 and 3.1 °C, respectively (in Fig. 3). In 450ppmeRCP, emissions are reduced even in 2020, with an ongoing reduction throughout the entire century. The emissions in 2100 are 9 GtCO_2_eq/year. In 450ppmeCancunP, the emissions reduction is delayed compared to 450ppmeRCP, with a value of 55 GtCO_2_eq/year in 2020. From 2020, this scenario shifts to a stage of drastic reductions, and the final emissions are lower than those in 450ppmeRCP in 2100 (2 GtCO_2_eq/year). In 450ppmeRCP and 450ppmeCancunP the emissions are similar in the middle of the 2020s. In 450ppmeINDC the emissions are reduced by half from 2030 to 2050, and are deeper than in the other scenarios in the latter half of the century. The scenario results in almost zero emissions in 2100. This scenario does not result in large negative CO_2_ emissions; however, if independent CO_2_ emissions are the focus, the negative CO_2_ emissions would be large (this information is shown in a later section).

In 2030, the emissions in 450ppmeINDC are around 15 GtCO_2_eq/year larger than in 450ppmeRCP, and therefore these additional emissions until 2030 need to be compensated for during the latter period. The global change in mean temperature in the three 450 ppm CO_2_-equivalent stabilization scenarios is around 1.7 °C at the end of the century (to maintain the >66 % probability, the average change in temperature is 1.7 rather than 2.0 °C). From 2030 to 2050, 450ppmINDC shows a rapid emissions reduction, with the annual reduction rate reaching 5 % per year. This value is quite high and would present difficulties for medium-term mitigation based on the INDCs.

### Primary energy supply and final energy consumption

**Fig. 4:**
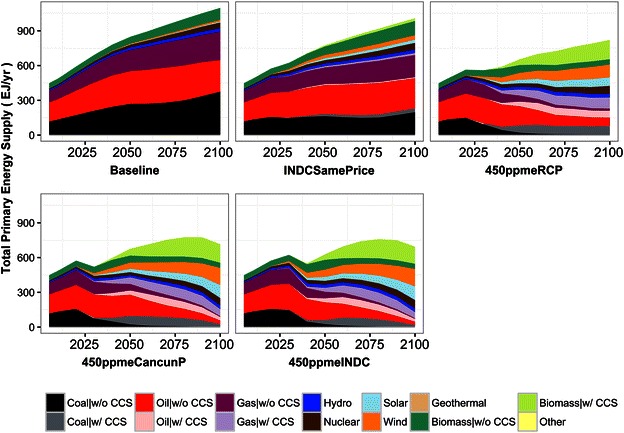
Global primary energy supply and its composition

Figure 4 shows the global primary energy supply for each scenario. At baseline, the total primary energy supply is the largest at around 1020 EJ/year in 2100, which is 2.3 times the current level (in 2005). In INDCSamePrice, the total primary energy supply in 2100 is around 10 % less than at baseline (930 EJ/year) due to energy savings in the energy end-use sectors and the shift in power generation from fossil fuel to renewable energy sources (the transformation efficiency is high in renewable energy). In the 450 ppm stabilization scenarios (450ppmeRCP, 450ppmeCancunP, and 450ppmeINDC), the energy supply is much smaller than at baseline at 730, 700, and 630 EJ/year in 2100, respectively. This decrease is realized by the large-scale saving of energy in energy end-use sectors and the transformation of power-generation sources. Among the three 450 ppm scenarios, the total energy in 2100 differs because of the timing of the drastic emissions reduction. As the timing of the reduction is delayed, the total energy supply in 2100 becomes lower. In terms of the composition of energy sources, fossil fuel remains the dominant source of energy supply in the baseline scenario. Renewable energies such as biomass, wind, and solar become the main energy sources in the 450 ppm stabilization scenarios. Carbon capture and storage (CCS) is almost fully implemented in the remaining industries with large fossil fuel consumption, such as steel and power generation. In 2030, there are primary energy supply reductions in 450ppmeCuncunP and 450ppmeINDC, but the reasons for this differ. In 450ppmeCuncunP, it is due to both the rapid emissions reduction and higher GHG emissions price (shown in Fig. 6, although the differences in the 2030s are not apparent). In 450ppmeINDC, it is due to the INDC commitment and the strong emissions reduction by large emitters in OECD countries, with the global total primary energy production in 2030 being low as a result (the regional primary supply in 2030 is shown in the Additional file 1).

**Fig. 5:**
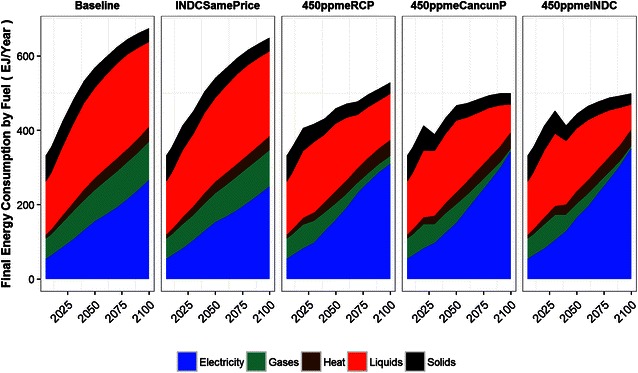
Global final energy consumption by fuels

The rapid emissions reduction in 450ppmeINDC and 450ppmeCancunP during the period from 2030 to 2050 also rely on final energy consumption side transformation. As illustrated in Fig. 5, total energy consumption drops rapidly during that period. Furthermore, regarding the fuel mix, fossil fuel oriented solid (mainly coal), liquid (mainly petroleum oil) and gases decrease whereas electricity increases.

### Mitigation cost

**Fig. 6:**
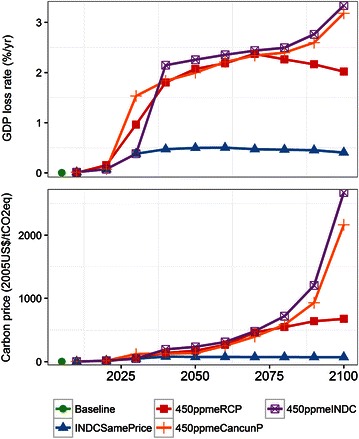
Global gross domestic product (GDP) loss rate and carbon prices

The mitigation cost (here we use GDP loss rates as a metric of mitigation cost) in INDCSamePrice is 0.4 % in 2030, and then is almost constant or slightly declines, as shown in Fig. 6. This is because the same carbon price is assumed after 2030, and the absolute cost of mitigation is kept almost the same, while GDP continuously grows. Comparing the three 450 ppm stabilization scenarios, 450ppmeRCP and 450ppmeCancunP, which have drastic emissions reductions by 2020, have high GDP loss rates in the early period. In contrast, 450ppmeINDC has a large GDP loss in the latter period. However, 450ppmeINDC and 450ppmeCancunP are not very different in the latter period. The GDP loss rate in 2050 is around 2.0 % for 450ppmeqRCP and 450ppmeCancunP, but is 2, 3.0, and 3.2 % in 2100 for 450ppmeRCP, 450ppmeCancunP, and 450ppmeINDC, respectively. The cost in 2100 is slightly lower than in IPCC AR5 (Clarke et al. 2014) but is almost the same in 2050.

**Fig. 7:**
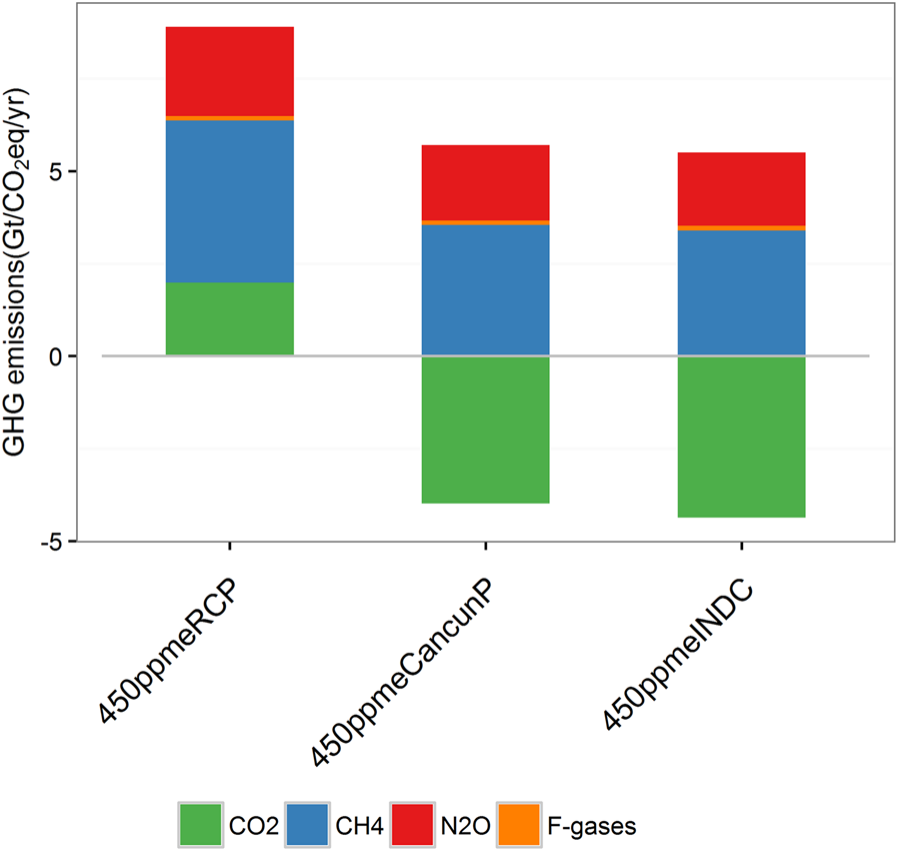
Greenhouse gas (GHG) emissions profile in 2100 for the 450 ppm stabilization scenarios

There are apparent differences in the carbon price between 450ppmeRCP and the other two 450 ppm stabilization scenarios (450ppmeINDC and 450ppmeCancunP). In 2100, 450ppmeRCP is around 650 $/tCO_2_eq, whereas 450ppmeINDC exceeds 3000 $/tCO_2_eq. In particular, because the marginal emissions reduction space in 450 ppmeINDC after 2070 is quite limited, the carbon price becomes much more sensitive to the incremental emissions reduction. Figure 7 shows the GHG emissions profile in 2100 for the 450ppm stabilization scenarios. The emissions of CH_4_, N_2_O, and F-gases are not very different among the three 450 ppm scenarios. This implies that there is limited scope for further non-CO_2_ emissions reduction in the high carbon price area. Under such conditions, the additional GHG emissions reduction must be realized by a CO_2_ emissions reduction. As a result, the total GHG emissions in 450 ppmeINDC and 450ppmeCancunP in the latter half of the century are around zero, and it is obvious that large-scale negative CO_2_ emissions are required. From the short-lived climate forcer (SLCF)’s point of view, CH4 is classified as SLCF and thought to play an important role for near-term climate policy aligning with air pollution policy. In 2030, 450ppmeRCP has a larger reduction in the near-term than other 4500ppm scenarios (Additional file 1).

### Land use and land-based CO_2_ emissions reduction

**Fig. 8:**
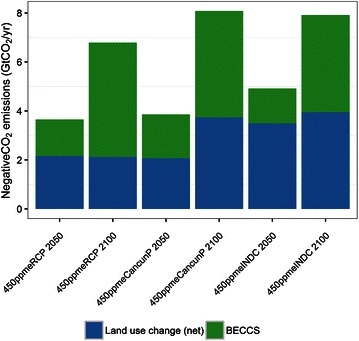
Negative CO_2_ emissions in 2050 and 2100

A negative CO_2_ emission can be realized only by biomass energy combined with CCS (BECCS) or afforestation in AIM/CGE. Although there is technology available that can directly capture CO_2_ from the atmosphere and store it underground (i.e., direct air capture), AIM/CGE does not consider it. Both BECCS and afforestation are measures that are strongly related to land use. Thus, we considered the CO_2_ sequestration achieved by BECCS and afforestation (Fig. 8). All of the 450 ppm scenarios have large negative CO_2_ emissions of more than 5 GtCO_2_eq/year in 2100. Remarkably, 450ppmeINDC and 450ppmeCancunP achieve 8 GtCO_2_/year in 2100, with a particularly pronounced increase in afforestation (4 GtCO_2_/year). This implies that around 300 Mha is required for afforestation, assuming a mean annual carbon sequestration of 3 tC/year/ha. Furthermore, bioenergy crops cover around 260 Mha in 2100 in 450ppmeINDC. The area used for land-based emissions-reduction measures eventually accounts for around 40 % of the current cropland area (1500 Mha). The differences in land use changes among models under various mitigation scenarios has been assessed previously (Popp et al. 2014). The area of cropland used for bioenergy in these scenarios is 24–36 % of the total cropland and our results are compatible with this figure.

## Discussion

Our results indicate that the 2 °C goal is achievable (in terms of a modeling exercise), even if the 2030s emissions are on track with the INDC targets. If the emissions in 2030 follow the INDCs, the emissions reduction after 2030 should be more drastic and several important characteristics are observed. First, a substantial and rapid emissions reduction is required from 2030 to 2050. Second, large-scale negative CO_2_ emissions and land-based CO_2_ emissions-reduction measures are required. The scale is more than that of 450ppmeRCP at the end of this century. Third, the carbon price becomes sensitive to the low GHG emissions area in the latter half of the century and the mitigation cost also increases.

Emissions should be drastically reduced after 2030 to achieve the 2 °C goal going through the INDC targets. This is more obvious in the mid-term until 2050 than in the latter period after 2050. This observation implies that the energy system would require rapid transformation. Gokul et al. (2015) reported that the additional energy system capacity could be huge with such rapid transformation. That is one of the aspects that we need to consider. Not only energy supply side, but also energy demand side showed rapid changes. Furthermore, the acceptability and capability of the social and economic system could be a major discussion point. AIM/CGE numerically provides a mathematical solution, but this does not necessarily mean that the real world can easily implement it, because the primary focus of AIM/CGE is to consistently foresee the long-term interaction of economic, energy, agriculture, and land use factors. In that sense, the model with the best or most detailed representation of energy technology and economic systems as well as energy demand side structure would be the most appropriate to use.

With regard to land use, there seems to be a requirement to consider a wide range of environmental concerns. If bioenergy crops need irrigation, water resources are placed under additional pressure. The ecosystems and biodiversity that would be affected by land use changes should be considered. If bioenergy crops require the application of nitrogen fertilizer, the nitrogen cycle would be affected and nitrogen pollution might become a concern. Large-scale afforestation would be implemented using productive species in terms of carbon sequestration, but such measures may limit the number of tree species and would influence the biodiversity and ecosystem balances. The AIM modeling framework has so far considered energy, monetary, and land use factors in the economic system, although water bodies, ecosystems, and other biophysical interactions are not explicitly taken into account. Thus, we need to incorporate them into the integrated framework. Some IAM studies, such as one on the Global Change Assessment Model (GCAM) (Kim et al. 2016), have already considered this.

The fact that the carbon price becomes sensitive in the low GHG emissions area implies that the emissions pathways in the latter half of the century should be carefully considered. In this study, we used the outputs of a DICE type intertemporal model, SCM4OPT, as the total GHG emissions constraint for AIM/CGE. However, we might be able to lessen the emissions reduction in the last few decades by a few Giga tons of CO_2_-equivalent emissions per year, to keep the carbon price and the mitigation cost relatively low and realistic, while the temperature increase would be close to 1.7 °C in 2100. Therefore, we should not interpret the results of this study as being the only emissions pathways to realize the 2 °C goal with INDCs. Rather, it would be better to be interpreted that the mitigation cost at the end of this century in INDCs is much larger than RCP2.6 to achieve 2 °C goal. Moreover, a very low level of GHG emissions is a necessary condition in the latter half of the century, which is similar to the RCP2.6 emissions pathways. More importantly, if the emissions in the first half of the century cannot be reduced as shown in this study, the carbon price would drastically increase. This also implies that numerically concrete emissions targets not only for 2030, but also for the mid-term, could be important to maintain the feasibility of long-term mitigation goals in the policy context relating to the PA. There is currently only a near-term, which is 2025–2030, emissions target and a long-term ultimate goal. A mid-term goal could help to achieve the long-term goal.

As discussed above, there are mid- and long-term difficulties to achieving the 2 °C goal based on meeting the INDC target, including rapid emissions reduction and negative CO_2_ emissions. However, this does not necessarily indicate that we should not aim to meet the 2 °C goal. First of all, the immediate policy reaction such as shown in 450ppmeRCP would reduce both med- and long-term difficulties. Second, it is obvious that the climate mitigation would certainly reduce the climate change impact. The economics of that benefit is presented in IPCC report (Arent et al. 2014). 3 °C increase which roughly corresponds to INDCSamePrice scenario could cause 2–3 % GDP loss whereas 1–2 °C increase would be almost zero impact. Considering these difficulties, we need to have a better assessment model and policy decisions that consider the impact of climate change and adaptation to its consequences. The climate change issue is complex in the sense that the polluters and victims differ temporally and spatially. A simple cost-benefit analysis would not provide the best solution. The uncertainty in the impact of climate change also makes the problem complex (IPCC 2014). However, the review and revision of NDCs in 2020 is a great opportunity to consider the above mitigation difficulties.

We have already discussed the current modeling limitations that need to be improved in the future. Here, we note three additional limitations of this study. First, the range of energy technologies considered in AIM/CGE could be expanded. One important technology that has not yet been incorporated is hydrogen. BECCS can be used in association with hydrogen fuel as a decarbonizing technology. For example, in a non-large point source but hard to decarbonize sector such as the transport sector, the use of hydrogen fuel would be an interesting research area. Second, we use only single climate sensitivity 3 °C, but if the climate sensitivity differs from the assumption made in this study, the conclusion might be affected. Considering a probability in climate model may help to get better understanding (Rogelj et al. 2013). In particular, by the end of this century, the carbon price is very sensitive to the emissions reduction requirement and slight changes in the climate response to the emissions reduction are clearly important. Third, although the global uniform carbon price is applied to all scenarios (after 2030 for 450ppmeINDC and after 2020 for 450ppmeCancunP) as is often done in many IAM studies, this is obviously an idealized modeling exercise and real policy implementation would be difficult.

## Conclusions

This study assessed long-term climate mitigation scenarios, to meet the 2 °C goal, considering INDCs. If the emissions in 2030 are as indicated in the INDCs, the emissions reduction in 2030–2050 need to be quite drastic, and large negative emissions in the latter half of century will be required to meet the 2 °C goal. We confirmed that there are mid-term and long-term difficulties to achieve the 2 °C goal with INDCs. To avoid them, additional reduction targets are an attractive option when counties review and revise their NDCs in 2020. Moreover, the emissions reduction commitments in the following decades (i.e., 2040s or 2050s) are also important to achieve the 2 °C goal. On the other hand, the current AIM framework does not sufficiently represent the fundamental elements and need to better incorporate land-related aspects, such as water bodies and ecosystems, as well as a better representation of energy technologies and economic systems for the mid-term assessment.

## Additional file

**Additional file 1.** Supplemental methodological description and results figures.

## Abbreviations

AIM: Asia-Pacific Integrated Model
INDCs: intended nationally determined contributions
COP: conference of the Parties
UNFCCC: United Nations Framework Convention on Climate Change
PA: Paris Agreement
NDCs: nationally determined contributions
MAC: marginal abatement cost
CGE: computable general equilibrium
AIM/PLUM: Asia-Pacific Integrated Model/integration Platform for Land-Use and Environmental Modeling
MAGICC: model for the assessment of greenhouse-gas induced climate change
CES: constant elasticity substitution
LES: linear expenditure system
IPCC AR5: fifth assessment report of intergovernmental panel on climate change
LPJmL: Lund-Potsdam-Jena managed Land Dynamic Global Vegetation and Water Balance Model
VISIT: vegetation integrative simulator for trace gases
SCM4OPT: simple climate model for optimization
RCP: representative concentration pathways
SSP: shared socioeconomic pathways
BECCS: biomass energy combined with CCS

## References

[CR1] Arent D, Tol R, Faust E, Hella J, Kumar S, Strzepek KM, Field CB, Barros VR, Dokken DJ, Mach KJ, Mastrandrea MD, Bilir TE, Chatterjee M, Ebi KL, Estrada YO, Genova RC, Girma B, Kissel ES, Levy AN, MacCracken S, Mastrandrea PR, White LL (2014). Key economic sectors and services. Climate change 2014: impacts, adaptation, and vulnerability. Part A: Global and sectoral aspects. Contribution of working group II to the fifth assessment report of the intergovernmental panel on climate change.

[CR2] Benveniste H, Criqui P, Boucher O, Breon F-M, Guivarch C, Prados E et al (2015) The INDC counter, aggregation of national contributrions and 2°C trajectories. http://hal.univ-grenoble-alpes.fr/hal-01245354

[CR3] Bondeau A, Smith PC, Zaehle S, Schaphoff S, Lucht W, Cramer W (2007). Modelling the role of agriculture for the 20th century global terrestrial carbon balance. Glob Change Biol.

[CR4] Clarke L, Jiang K, Akimoto K, Babiker M, Blanford G, Fisher-Vanden K et al (2014) Assessing transformation pathways. In: Climate change 2014: mitigation of climate change. Contribution of working group III to the fifth assessment report of the intergovernmental panel on climate change. Cambridge University Press, Cambridge

[CR5] Climate Action Tracker (2015) Tracking INDCs. http://climateactiontracker.org/. Accessed 2 Feb 2016

[CR6] Fawcett AA, Iyer GC, Clarke LE, Edmonds JA, Hultman NE, McJeon HC (2015). Can Paris pledges avert severe climate change?. Science.

[CR01] Fujimori S, Masui T, Matsuoka Y (2012) AIM/CGE [basic] manual, Discussion paper series. Center for Social and Environmental Systems Research, National Institute Environemntal Studies, pp 1–87

[CR7] Fujimori S, Hasegawa T, Masui T, Takahashi K (2014). Land use representation in a global CGE model for long-term simulation: CET vs. logit functions. Food Secur.

[CR8] Fujimori S, Kainuma M, Masui T, Hasegawa T, Dai H (2014). The effectiveness of energy service demand reduction: a scenario analysis of global climate change mitigation. Energy Policy.

[CR9] Fujimori S, Masui T, Matsuoka Y (2014). Development of a global computable general equilibrium model coupled with detailed energy end-use technology. Appl Energy.

[CR10] Fujimori S, Masui T, Matsuoka Y (2015). Gains from emission trading under multiple stabilization targets and technological constraints. Energy Econ.

[CR36] Fujimori S, Hasegawa T, Masui T, Takahashi K, Herran DS, Dai H, Hijioka Y, Kainuma M (2016) SSP3: AIM implementation of shared socioeconomic pathways. Global Environmental Change. doi:10.1016/j.gloenvcha.2016.06.009

[CR11] Gokul CI, James AE, Allen AF, Nathan EH, Jameel A, Ghassem RA (2015). The contribution of Paris to limit global warming to 2 & #xB0;C. Environ Res Lett.

[CR37] Hasegawa T, Fujimori S, Takahashi K, Ito A, Masui T (under review) Land use allocation model development and its application in AIM. PLOSONE

[CR12] Hasegawa T, Fujimori S, Shin Y, Takahashi K, Masui T, Tanaka A (2014). Climate change impact and adaptation assessment on food consumption utilizing a new scenario framework. Environ Sci Technol.

[CR13] Hasegawa T, Fujimori S, Shin Y, Tanaka A, Takahashi K, Masui T (2015). Consequence of climate mitigation on the risk of hunger. Environ Sci Technol.

[CR14] Hasegawa T, Fujimori S, Takahashi K, Masui T (2015). Scenarios for the risk of hunger in the twenty-first century using shared socioeconomic pathways. Environ Res Lett.

[CR15] Hasegawa T, Fujimori S, Takahashi K, Yokohata T, Masui T (2016). Economic implications of climate change impacts on human health through undernourishment. Clim Change.

[CR16] Hof A, van Soest H, van den Berg M, de Boer HS, den Elzen M, Harmsen M et al (2015) Raising the ambition level of INDCs allows for a smoother energy transition. Assessment of the implications of INDCs for achieving the 2°C climate goal.: PBL Netherlands Environmental Assessment Agency

[CR17] International Energy Agency (IEA) (2015) World energy outlook special report 2015: energy and climate change. International Energy Agency

[CR18] International Institute for Applied Systems Analysis (IIASA) (2015a) IAMC AR5 scenario database. https://secure.iiasa.ac.at/web-apps/ene/AR5DB/dsd?Action=htmlpage&page=about#intro

[CR19] International Institute for Applied Systems Analysis (IIASA) (2015b) Shared socioeconomic pathways (SSP) database version 0.9.3. https://secure.iiasa.ac.at/web-apps/ene/SspDb

[CR20] IPCC (2007) Climate change 2007: the physical science basis. Contribution of working group I to the fourth assessment report of the intergovernmental panel on climate change

[CR21] IPCC (2014) IPCC, 2014: climate change 2014: synthesis report. contribution of working groups I, II and III to the fifth assessment report of the intergovernmental panel on climate change. IPCC

[CR22] Ito A, Inatomi M (2012). Water-use efficiency of the terrestrial biosphere: a model analysis focusing on interactions between the global carbon and water cycles. J Hydrometeorol.

[CR23] Kim SH, Hejazi M, Liu L, Calvin K, Clarke L, Edmonds J (2016). Balancing global water availability and use at basin scale in an integrated assessment model. Clim Change.

[CR24] Kitous A, Keramidas K (2015) Analysis of scenarios integrating the INDCs. European comission

[CR25] Lomborg B (2015). Impact of current climate proposals. Global Policy.

[CR26] Meinshausen M, Raper SCB, Wigley TML (2011). Emulating coupled atmosphere-ocean and carbon cycle models with a simpler model, MAGICC6—part 1: model description and calibration. Atmos Chem Phys.

[CR27] Meinshausen M, Jeffery L, Guetschow J, Robiou du Pont Y, Rogelj J, Schaeffer M et al (2015) National post-2020 greenhouse gas targets and diversity-aware leadership. Nat Clim Change 5(12):1098–1106. doi:10.1038/nclimate2826. http://www.nature.com/nclimate/journal/v5/n12/abs/nclimate2826.html#supplementary-information

[CR28] Mittal S, Dai H, Fujimori S, Masui T (2016). Bridging greenhouse gas emissions and renewable energy deployment target: comparative assessment of China and India. Appl Energy.

[CR29] Nordhaus W, Sztorc P (2013) DICE 2013R: introduction and user’s manual, second edition. http://www.econ.yale.edu/~nordhaus/homepage/documents/DICE_Manual_103113r2.pdf

[CR30] Popp A, Rose SK, Calvin K, Van Vuuren DP, Dietrich JP, Wise M (2014). Land-use transition for bioenergy and climate stabilization: model comparison of drivers, impacts and interactions with other land use based mitigation options. Clim Change.

[CR31] Rogelj J, McCollum DL, Reisinger A, Meinshausen M, Riahi K (2013). Probabilistic cost estimates for climate change mitigation. Nature.

[CR32] Rosenzweig C, Elliott J, Deryng D, Ruane AC, Müller C, Arneth A (2014). Assessing agricultural risks of climate change in the 21st century in a global gridded crop model intercomparison. Proc Natl Acad Sci.

[CR33] Schumacher K, Sands RD (2006). Innovative energy technologies and climate policy in Germany. Energy Policy.

[CR34] United Nations Framework Convention on Climate Change (UNFCCC) (2015) Adoption of the Paris agreement. Proposal by the president (1/CP21). http://unfccc.int/resource/docs/2015/cop21/eng/10a01.pdf. Accessed 2 Feb 2016

[CR35] Vuuren DP, Stehfest E, Elzen MGJ, Kram T, Vliet J, Deetman S (2011). RCP2.6: exploring the possibility to keep global mean temperature increase below 2°C. Clim Change.

