# Erratum to: Implication of Paris Agreement in the context of long-term climate mitigation goals

**DOI:** 10.1186/s40064-017-3787-3

**Published:** 2017-05-31

**Authors:** Shinichiro Fujimori, Xuanming Su, Jing-Yu Liu, Tomoko Hasegawa, Kiyoshi Takahashi, Toshihiko Masui, Maho Takimi

**Affiliations:** 1 Center for Social and Environmental Systems Research, National Institute for Environmental Studies (NIES), National Institute for Environmental Studies, 16-2 Onogawa, Tsukuba, Ibaraki 305–8506 Japan; 2 International Institute for Applied Systems Analysis, Schlossplatz-1, 2361 Laxenburg, Austria; 3 Mizuho Information and Research Institute, Inc., 2-3 Kanda-Nishikicho, Chiyoda-ku, Tokyo 101-8443 Japan

## Erratum to: SpringerPlus (2016) 5:1620 DOI 10.1186/s40064-016-3235-9

After production of the original article (Fujimori et al. 2016) the author of reference 13, which currently reads “Gokul CI, James AE, Allen AF, Nathan EH, Jameel A, Ghassem RA et al (2015) The contribution of Paris to limit global warming to 2 & #xB0;C. Environ Res Lett 10(12):125002”, contacted the author of the paper published in SpringerPlus and asked to correct the reference so that it reads “Iyer, G.C., Edmonds, J.A., Fawcett, A.A., Hultman, N.E., Alsalam, J., Asrar, G.R., Calvin, K.V., Clarke, L.E., Creason, J., Jeong, M. and Kyle, P., 2015. The contribution of Paris to limit global warming to 2 °C. Environmental Research Letters, 10(12), p. 125002”.

This correction request has been acknowledged by means of this erratum.
