# Supplementary material for: Implication of Paris Agreement in the context of long-term climate mitigation goals
Source: Springerplus. 2016 Sep 20;5(1):1620. doi: 10.1186/s40064-016-3235-9 (PMC5028350; doi:10.1186/s40064-016-3235-9)
Supplement: Supplementary file 1 — Additional file 1. Supplemental methodological description and results figures. [file 40064_2016_3235_MOESM1_ESM.pdf]

Supporting Information for

Implication of Paris Agreement in the Context of Long-term Climate Mitigation Goal

1. Regional and sectoral resolution of the model 2
2. SCM4OPT 3
3. Emissions constraint for INDCs 6
4. Energy supply and power system in 2030 for INDCs and Baseline 7

1. Regional and sectoral resolution of the model

SI Table 1 Region classification

Code	Region	Code	Region
JPN	Japan	TUR	Turkey
CHN	China	CAN	Canada
IND	India	USA	United States
XSE	Southeast Asia	BRA	Brazil
XSA	Rest of Asia	XLM	Rest of South America
XOC	Oceania	XME	Middle East
XE25	EU25	XNF	North Africa
XER	Rest of Europe	XAF	Rest of Africa
CIS	Former Soviet Union		

SI Table 2 Industrial classification

Agricultural sectors	Energy supply sectors	Other production sectors
Rice	Coal mining	Mineral mining and other quarrying
Wheat	Oil mining	Food products
Other grains	Gas mining	Textiles, apparel, and leather
Oil seed crops	Petroleum refinery	Wood products
Sugar crops	Coal transformation	Paper, paper products, and pulp
Other crops	Biomass transformation (1st generation)	Chemical, plastic, and rubber products
Ruminant livestock	Biomass transformation (2nd generation with energy crop)	Iron and steel
Raw milk	Biomass transformation (2nd generation with residue)	Nonferrous products
Other livestock and fisheries	Gas manufactures distribution	Other manufacturing
Forestry	Coal-fired power	Construction
	Oil-fired power	Transport and communications
	Gas-fired power	Other service sectors
	Nuclear power	CCS service
	Hydroelectric power	
	Geothermal power	
	Photovoltaic power	
	Wind power	
	Waste biomass power	
	Other renewable energy power generation	
	Advanced biomass-power generation	

2. SCM4OPT

1) Extend the simple climate model

We introduced the carbon cycle, physical processes for simulating the concentration, and forcing for each emission based on MAGICC 6.0 (Meinshausen et al. 2011), and used a simplified temperature module to generate the temperature increase above the preindustrial level, avoiding the complexities resulting from the upwelling-diffusion climate model. Therefore, the temperature increase could feedback into socioeconomic development in the optimization process.

a. The carbon cycle in MAGICC 6.0 was introduced for a more precise depiction of the formation of the CO2 concentration in the atmosphere. For the terrestrial carbon cycle, the carbon fluxes among the atmosphere, living plants, detritus, and soil were considered and simulated separately. The perturbation of the ocean surface dissolved inorganic carbon in the ocean carbon cycle was modeled by an impulse response function (Joos et al. 2001) with consideration of the sensitivity of the sea surface partial pressure to changes in temperature above the preindustrial level (Takahashi et al. 1993). All of the non-CO2 gases, including CH4, N2O, F-Gases, CO, VOC, SOx, NOx, BC, and OC, were simulated using similar physical processes as MAGICC 6.0. We calibrated the SCM4OPT with MAGICC 6.0 using all four RCPs, which made the SCM4OPT capable of evaluating a wide range of potential forcing, with respect to the uncertainty in future socioeconomic development. The calibration results were as follows:

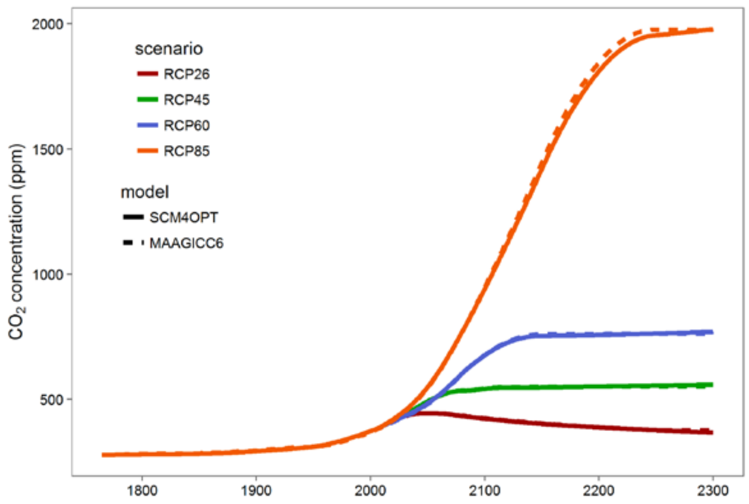

SI Figure 1 CO_2 concentration between SCM4OPT and MAGICC 6.0

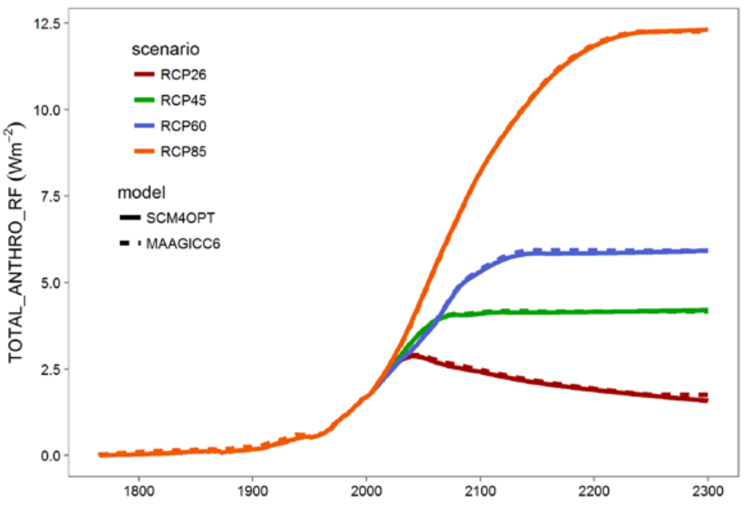

SI Figure 2 Total anthropogenic forcing

b. A simplified temperature module was used to simulate the temperature increase above the preindustrial level, resulting from human-induced or natural radiative forcing. A two-boxes model was built as in DICE2013R; however, we adjusted the standard radiative forcing $\Delta Q(t)$ to the effective radiative forcing $\Delta Q_e(t)$ by multiplying by an efficacy term, E_a:

$$\Delta Q_e(t) = E_a \cdot \Delta Q(t)$$

where t is the simulation time (years).

We used the adjusted effective radiative forcing in the two-boxes model to estimate the temperature increase above preindustrial levels, as shown in SI Figure 3.

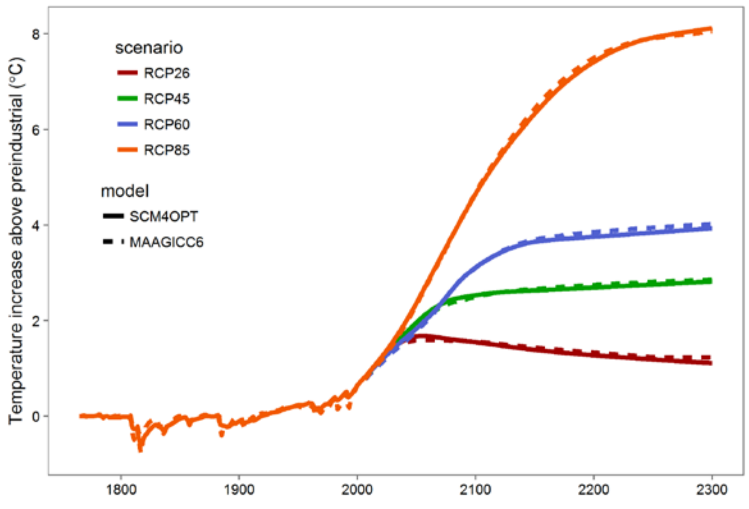

SI Figure 3 Temperature increase above the preindustrial level

2) Restructure DICE2013R
3) To model the SSPs, the population and GDP for each scenario were derived from the SSP quantified elements. Global-scale modeling factors, such as the industrial CO2 emission intensity and other emissions (CO2 emissions from land use, CH4, N2O, F-Gases, CO, VOC, SOx, NOx, black carbon [BC], and organic carbon [OC]) were adopted from the AIM/CGE baseline case output. We used two

groups of data generated from AIM/CGE for sensitivity analysis. Each group defined 10 carbon prices from 0 US$/t-CO2 in 2010, to 100 – 1000 US$/t-CO2 in 2100, with linear or exponential trends within the century. Then the marginal abatement cost (MAC) curve for each SSP was estimated using industrial CO2 emission control rates and carbon prices. The estimated MAC of SSP2 is shown in SI Figure 4.

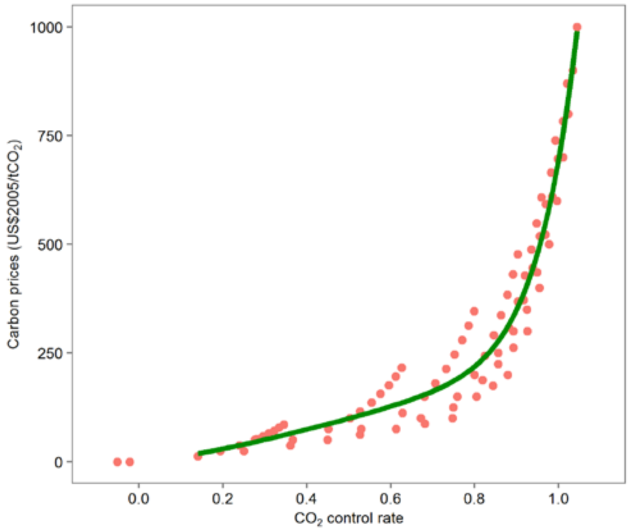

SI Figure 4 Estimation of MAC

As in DICE2013R, we used the industrial CO_2 control rate μ to represent potential future climate abatement options:

$$E_{ind} = YG \cdot \sigma \cdot (1 - \mu)$$

where E_{ind} is the level of industrial CO_2 emissions after emissions control, YG is the gross output, and σ denotes the intensity of industrial CO_2 emissions.

The definitions of other economic indicators and relationships were similar to those in DICE2013R, which maximizes social welfare by balancing the costs of climate change and potential future climate damage.

3. Emissions constraint for INDCs

We have taken the INDC information from the webpage(United Nations Frameowrk Convention on Climate Change 2016) and translated into the emissions constraints in the model. Basically, we made the 2030's emissions target and then linearly connected with 2020's emissions. For those countries declaring the target year as 2025 (e.g. US), we made the emissions constraint for 2025 at first and then, calculate emissions reduction rate from 2020 to 2025, and finally, adopt that reduction rate from 2025 to 2030.

There are ten types of commitment as shown SI Table 3. The emissions coverage is GHG or CO_2 and some countries use emissions intensity. The reference diverges the year from 1990 to 2014 and moreover, the baseline is also used by some countries. In case using reference year before 2005 which is the base year of the model simulation, we use EDGAR4.2 emissions inventory to determine the emissions target. For those countries which use the year after 2005 as the reference, we use the emissions results in the baseline scenario. The GDP in 2030 is used for the intensity cases. There are some counties which use specific sector's emissions target, but we ignore such very special case because it is hard to implement in model analysis and they account for a tiny proportion in global total emissions. If countries are treated as a single region in the model (like Japan and China), there is no problem for case 7 because we can obtain the identical baseline scenario. However, if the countries are aggregated into a region (e.g. Rest of Asia), we need to derive baseline emissions for such countries. In order to do, assuming that we have GDP assumptions for every country, we used the baseline scenario's emissions intensity change in the aggregated region. Then, GDP and emissions intensity change of each country can derive the emissions in the baseline scenario.

SI Table 3 List of INDC commitment patterns

Case	Emissions	Reference	Data source and assumption
1	GHG Emissions	1990	Based on EDGAR4.2 (EC-JRC/PBL 2012)
2	GHG Emissions	1994	
3	GHG Emissions	2000	
4	GHG Emissions	2005	
5	GHG Emissions	2010	Based on emissions in the reference year of baseline scenario and GDP in 2030
6	GHG Emissions	2014	
7	GHG Emissions	baseline	Based on baseline scenario
8	GHG Emissions intensity	2005	Based on EDGAR4.2 (EC-JRC/PBL 2012) and GDP in 2030
9	CO2 Emissions intensity	2005	
10	GHG Emissions intensity	2007	Based on baseline scenario
11	GHG Emissions intensity	2010	
12	GHG Emissions intensity	baseline	

4. Energy supply and power system in 2030 for INDCs and Baseline

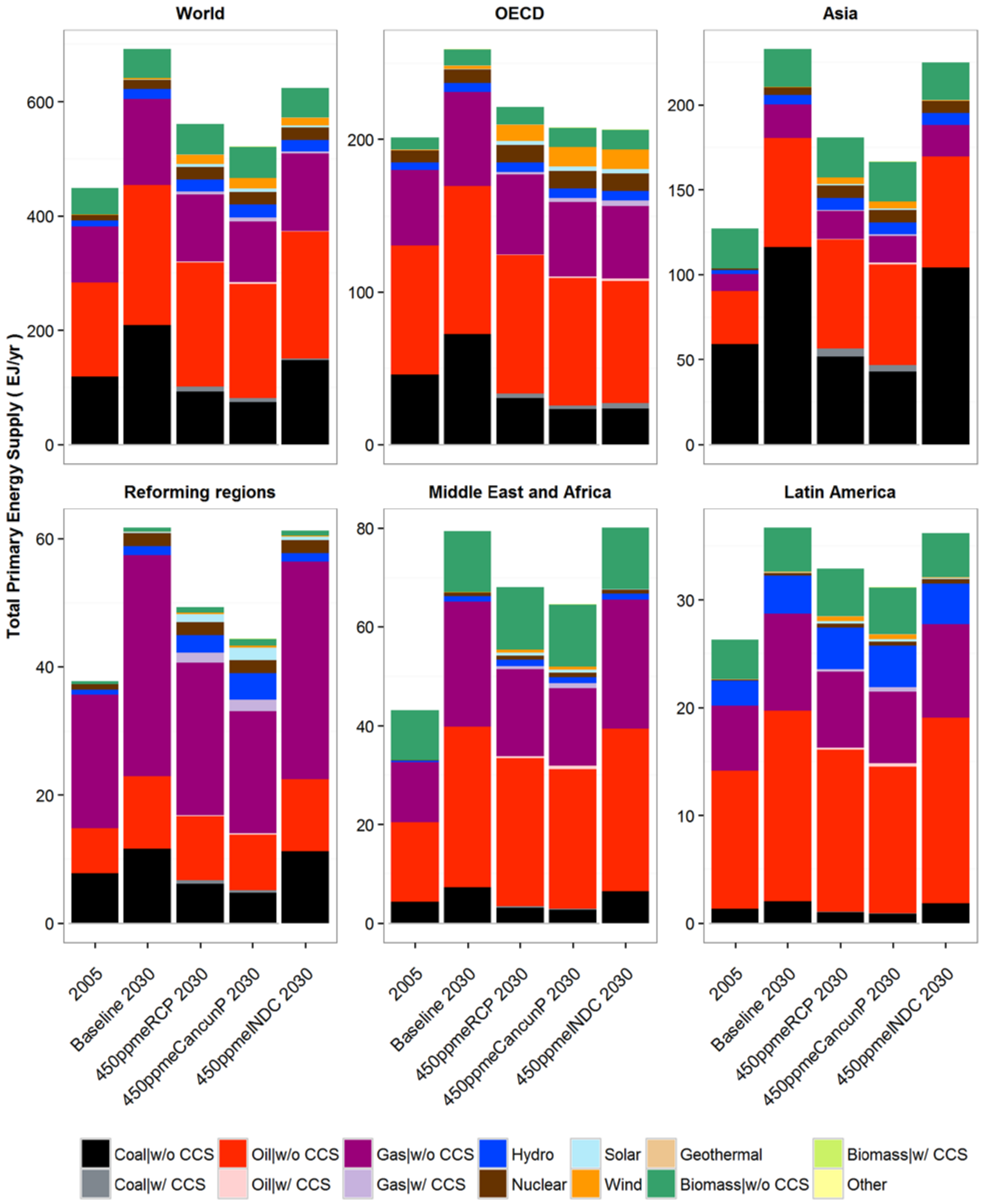

SI Figure 5 Primary energy supply by energy sources for aggregated five regions in 2005 and 2030.

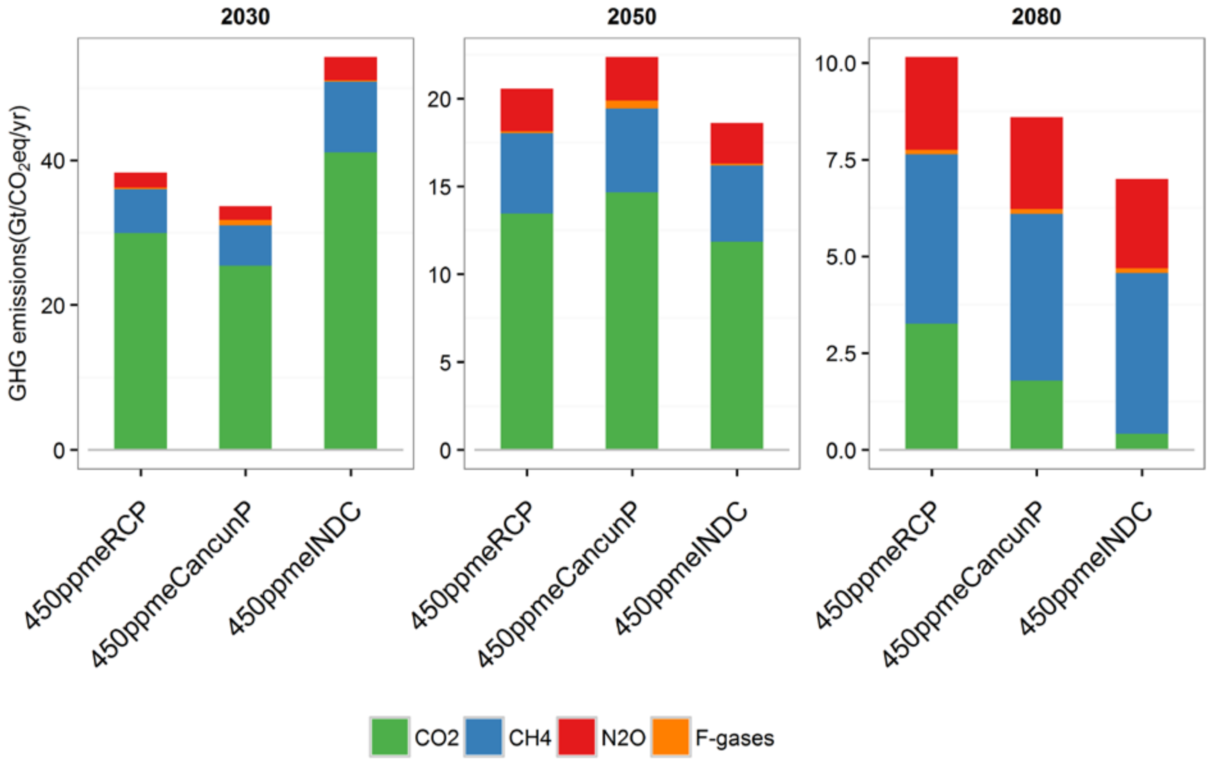

SI Figure 6 GHG emissions in 2030, 2050 and 2080.

References

EC-JRC/PBL (2012) Emission Database for Global Atmospheric Research (EDGAR), release version 4.2. http://edgar.jrc.ec.europa.eu.

Joos F, Prentice IC, Sitch S, Meyer R, Hooss G, Plattner G-K, Gerber S, Hasselmann K (2001) Global warming feedbacks on terrestrial carbon uptake under the Intergovernmental Panel on Climate Change (IPCC) Emission Scenarios. Global Biogeochemical Cycles 15 (4):891-907. doi:10.1029/2000GB001375

Meinshausen M, Raper SCB, Wigley TML (2011) Emulating coupled atmosphere-ocean and carbon cycle models with a simpler model, MAGICC6 – Part 1: Model description and calibration. Atmos Chem Phys 11 (4):1417-1456. doi:10.5194/acp-11-1417-2011

Takahashi T, Olafsson J, Goddard JG, Chipman DW, Sutherland SC (1993) Seasonal variation of CO2 and nutrients in the high-latitude surface oceans: A comparative study. Global Biogeochemical Cycles 7 (4):843-878. doi:10.1029/93GB02263

United Nations Frameowrk Convention on Climate Change, (UNFCCC) (2016) INDCs as communicated by Parties. http://www4.unfccc.int/submissions/indc/Submission%20Pages/submissions.aspx.
